# Supplementary material for: Discovery and pharmacophoric characterization of chemokine network inhibitors using phage-display, saturation mutagenesis and computational modelling
Source: Nat Commun. 2023 Sep 16;14:5763. doi: 10.1038/s41467-023-41488-z (PMC10505172; doi:10.1038/s41467-023-41488-z)
Supplement: Supplementary file 1 — Supplementary Information [file 41467_2023_41488_MOESM1_ESM.pdf]

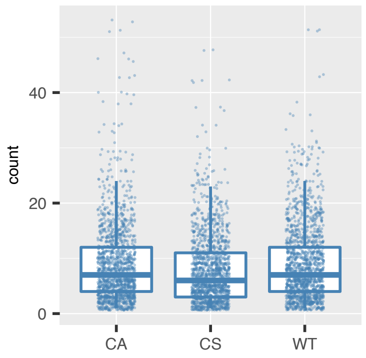

Supplementary Figure 1. Effect of Cys to Ala or Cys to Ser mutation on library construction. Box-whisker plot showing effect of mutation on peptide counts in the phage-display library. Y-axis shows counts obtained by next generation sequencing of individual peptides in peptide classes CA (Cys to Ala mutant, n = 1586), CS (Cys to Ser mutant, n= 1585) and WT (wild-type, n = 1607). Individual data points are shown. The box-whisker plot shows the median as centre, 25th and 75th percentile as bounds, and 1.5*interquartile range as whiskers.

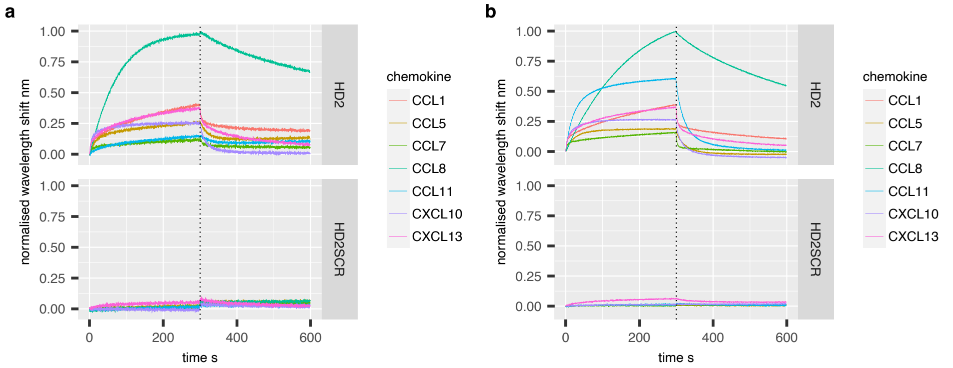

Supplementary Figure 2. Binding of chemokines to HD2 and HD2SCR. a, b, Faceted plots of biolayer interferometry (BLI) sensorgrams showing cross-binding of SUMO:HD2 and SUMO:HD2SCR to human chemokines from two independent experiments. Plots display normalised wavelength shift (y axis; nm) versus time (x-axis; seconds). Vertical dotted line indicates the onset of dissociation. Cross-binding experiments (at 1 uM chemokine concentration) were performed with a panel of chemokines (see Supplementary information Table 3). Specific binders were identified where Rmax (i.e. normalised wavelength shift at 10 s before the end of association) obtained with SUMO:HD2 exceeded 3 times the Rmax obtained with SUMO:HD2SCR. Chemokine names are indicated. Only chemokines that showed reproducible and specific binding in two independent experiments are shown.

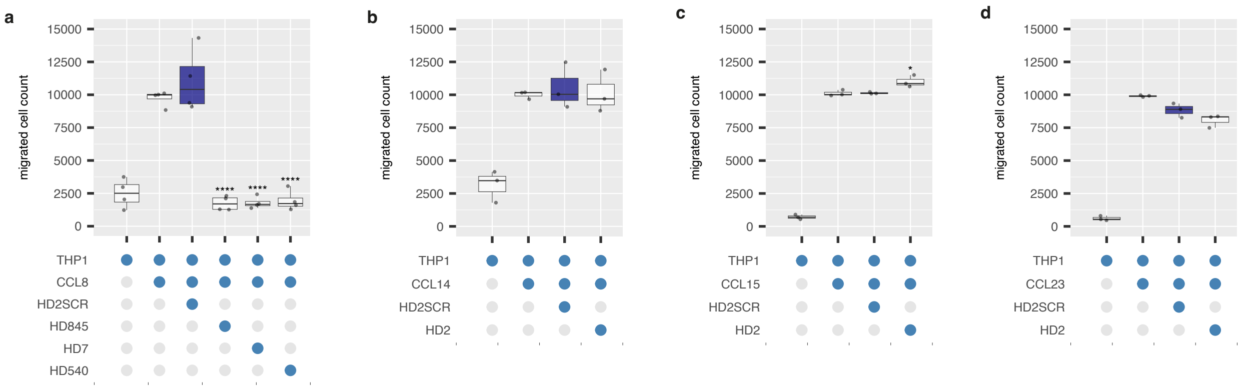

Supplementary Figure 3. Effect of exemplar peptides on chemotaxis. a-d, Box-whisker plots showing the effect of the indicated peptides on cell migration induced by indicated human chemokines. All experiments were performed as three technical and three biological replicates, and individual biological replicate data points (mean of technical replicates) are shown. Y-axis in each panel shows migrated cell count normalized to the median value of migrated cells in the presence of chemokine alone, set at 10000 cells. X-axis shows constituents of each experiment as blue-filled dots. Chemokine names are indicated. HD2SCR is a scrambled version of HD2 used as negative control. All peptides were at 10 uM final concentration and chemokines at EC80 doses. Each box-whisker plot shows the median as centre, 25th and 75th percentile as bounds, and 1.5*interquartile range as whiskers. Statistically significant differences (compared to control, coloured blue), using a two-sided Dunnett's test with correction for multiple comparisons, are indicated by asterisks: **** = $P \leq 0.0001$, *** = $P \leq 0.001$, ** = $P \leq 0.01$, * = $P \leq 0.05$, n= 3 biological replicates in each group. Exact P-values for panel **a** HD845, HD7 and HD540 are: P=0.00000.

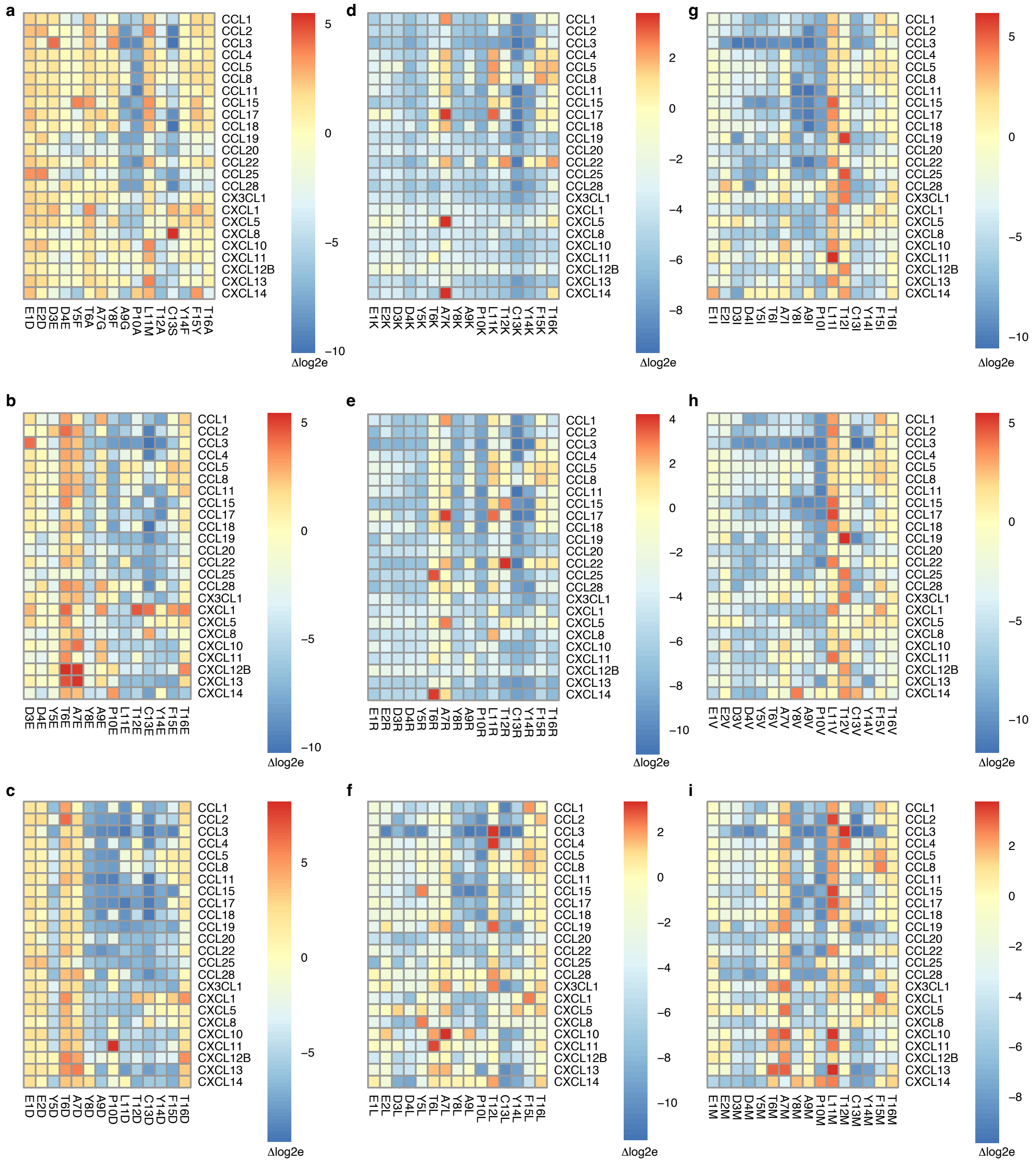

Supplementary Figure 4. **Effect of HD2 mutations on phage binding.** Tileplots of HD2 mutations with tile colour showing Δlog2E, which is the difference in log2E between the parental wild-type peptide and the mutant variant following phage-display selection. Rows show the selecting chemokine and columns the mutation. Scale bar shows Δlog2E values. **a,** Conservative residue substitutions **b,c,** Anionic residue substitutions. **d,e,** Cationic residue substitutions. **f-i,** Hydrophobic residue substitutions.

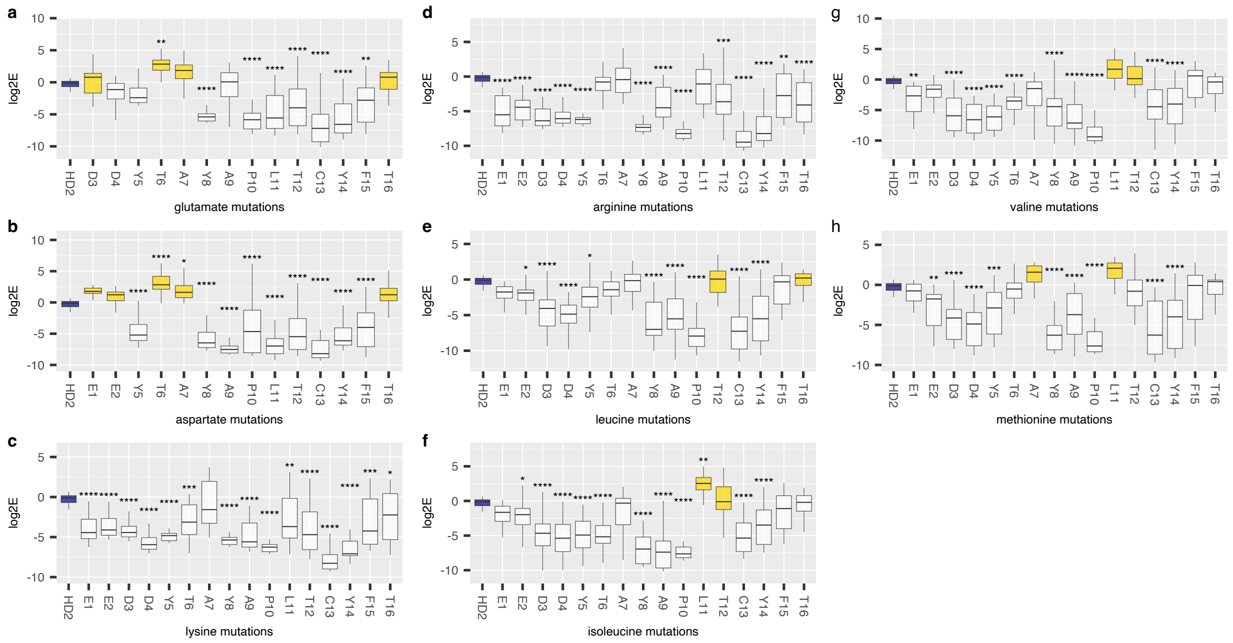

Supplementary Figure 5. Effect of HD2 mutations on phage binding. Box-whisker plots showing impact of HD2 residue substitutions by glutamic acid (**a**), aspartic acid (**b**), lysine (**c**), arginine (**d**), leucine (**e**), isoleucine (**f**), valine (**g**) and methionine (**h**) residues respectively (X-axis) upon log2E (Y-axis). Each box-whisker plot shows the median as centre, 25th and 75th percentile as bounds, and 1.5*interquartile range as whiskers. Statistically significant differences (compared to control, coloured blue), using a two-sided Dunnett's test with correction for multiple comparisons, are indicated by asterisks: **** = $P \leq 0.0001$, *** = $P \leq 0.001$, ** = $P \leq 0.01$, * = $P \leq 0.05$, n= 24 per group. Exact P values are provided in Supplementary Table 5. Boxes showing a positive value for difference from control (identified from Dunnett's test) are shown as yellow.

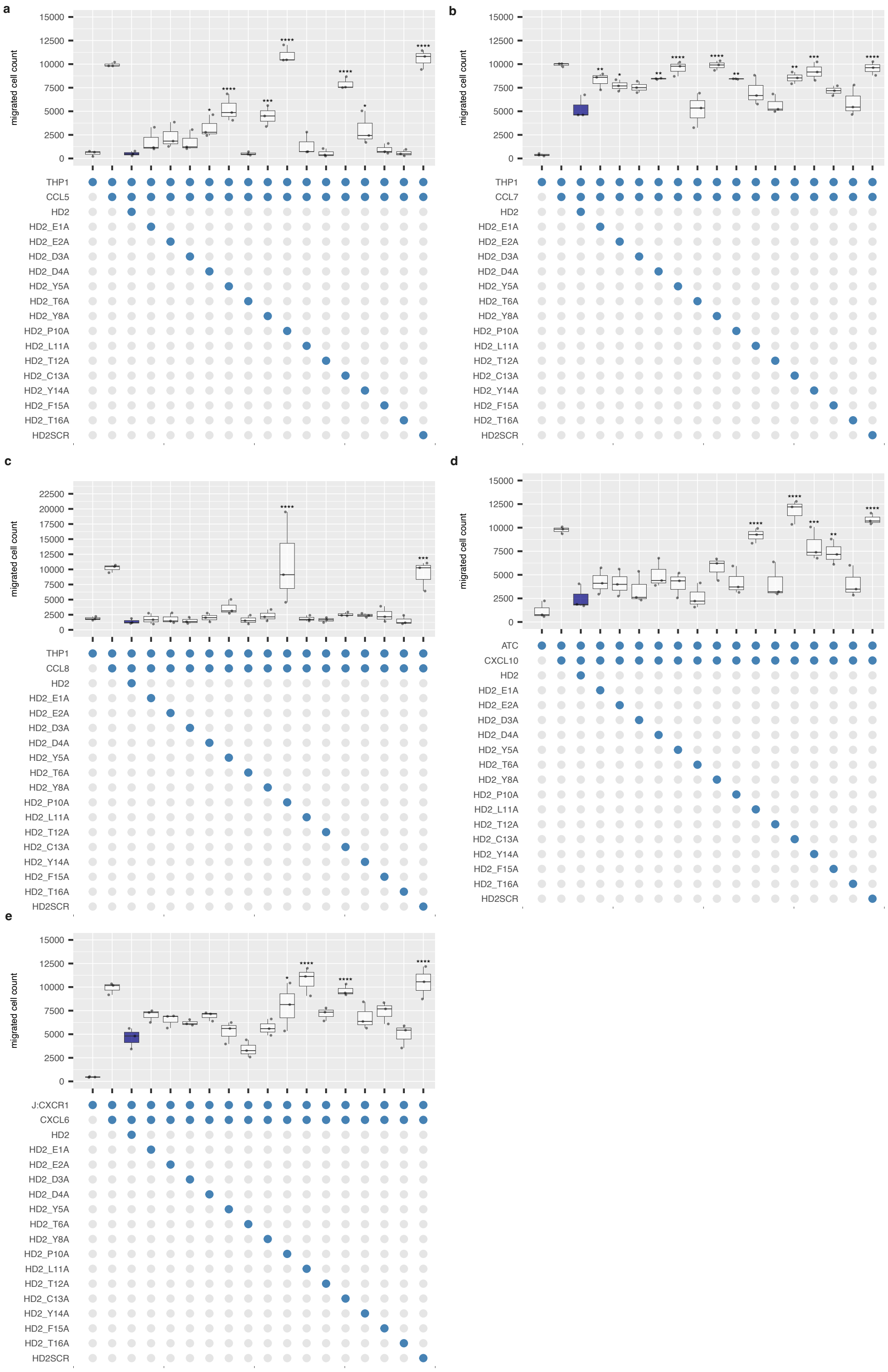

Supplementary Figure 6. Effect of HD2 alanine mutations on chemotaxis. a-e, Box-whisker plots showing the effect of the indicated alanine mutant peptides on cell migration induced by indicated human chemokines. All experiments were performed as three technical and three biological replicates, and individual biological replicate data points (mean of technical replicates) are shown. Y-axis in each panel shows migrated cell count normalized to the median value of migrated cells in the presence of chemokine alone, set at 10000 cells. X-axis shows constituents of each experiment as blue-filled dots. Chemokine names are indicated. HD2SCR is a scrambled version of HD2. All peptides were at 10 uM final concentration and chemokines at EC80 doses. Each box-whisker plot shows the median as centre, 25th and 75th percentile as bounds, and 1.5*interquartile range as whiskers. Statistically significant differences (compared to parental peptide HD2, coloured blue), using a two-sided Dunnett's test with correction for multiple comparisons, are indicated by asterisks: **** = $P \leq 0.0001$, *** = $P \leq 0.001$, ** = $P \leq 0.01$, , * = $P \leq 0.05$, n= 3 biological replicates in each group. Exact P values are reported in Supplementary Table 6.

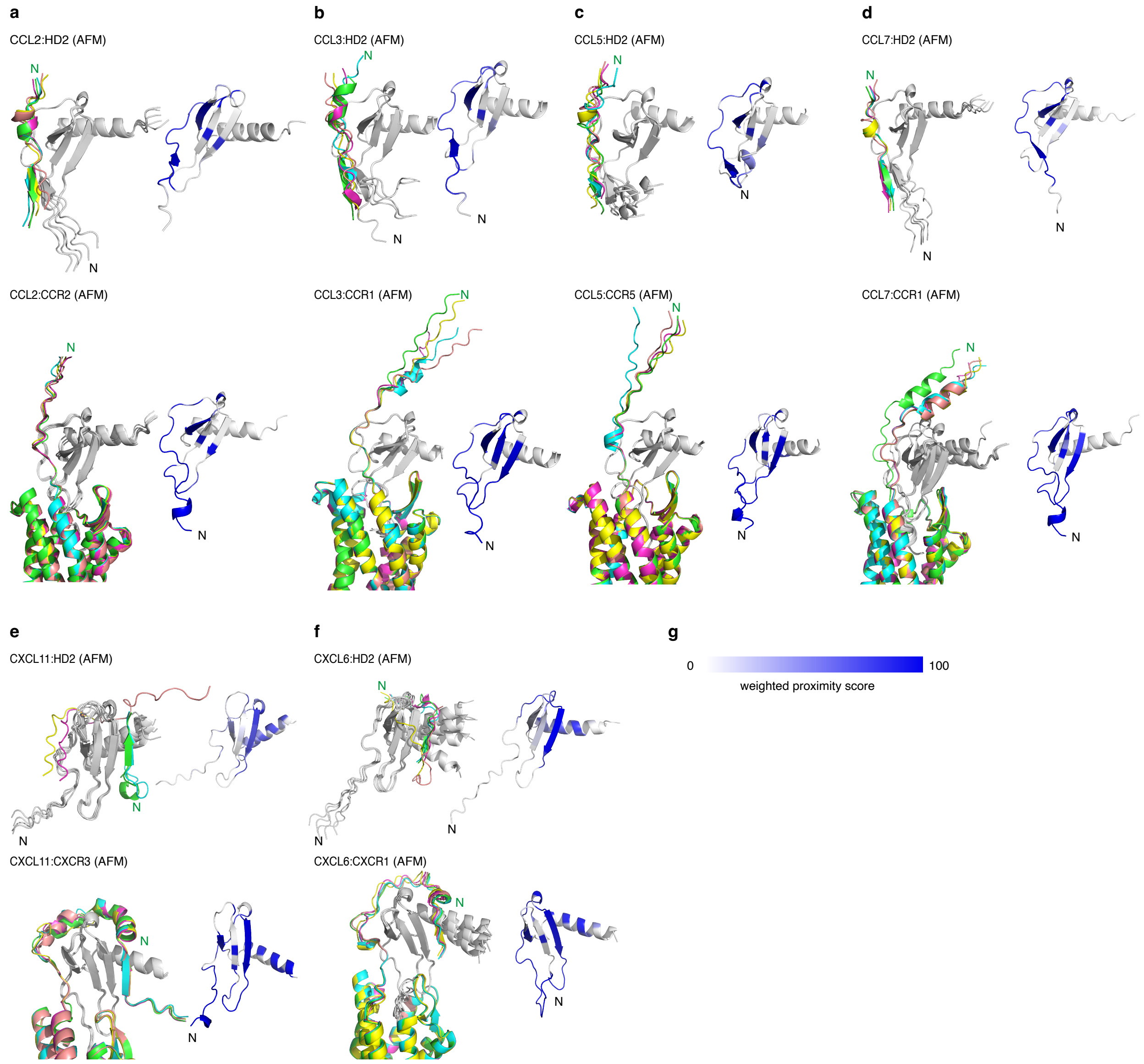

Supplementary Figure 7. Predicted binding modes of HD2 and receptors to chemokines using AlphaFold2-Multimer. a-f, *Left panels:* Ribbon diagrams showing predicted poses for chemokine:HD2 (*top*) and for chemokine:receptor (*bottom*). Chemokines are in gray, and the peptide or receptor in colour, with the top-ranked pose coloured green. *Right panels:* Corresponding heatmaps of weighted proximity scores mapped onto ribbon diagrams of the chemokine. **g,** Scale bar for weighted proximity score. *Abbreviation:* N: N-terminal residue of the top-ranked pose. Source data are provided as a Source Data file.

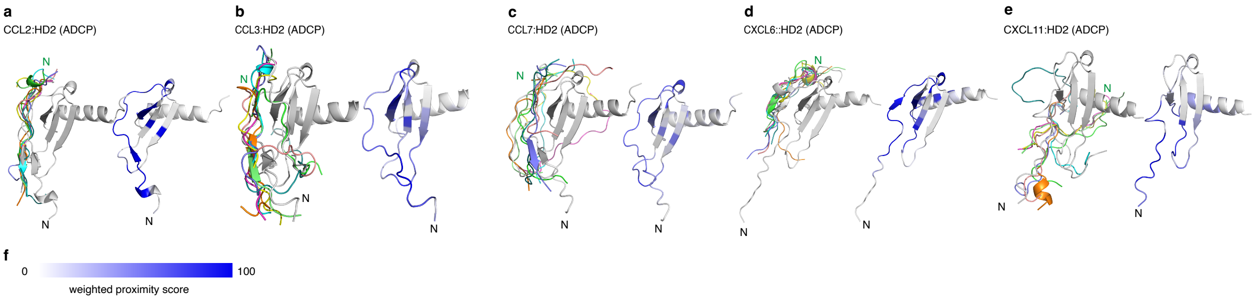

Supplementary Figure 8. Predicted binding modes of HD2 to chemokines using AutoDock CrankPep. a-e, *Left panels:* Ribbon diagrams showing predicted poses for chemokine. Chemokines are in gray, and the peptide in colour, with the top-ranked pose coloured green. *Right panels:* Corresponding heatmaps of weighted proximity scores mapped onto ribbon diagrams of the chemokine. **f,** Scale bar for weighted proximity score. *Abbreviation:* N: N-terminal residue of the top-ranked pose. Source data are provided as a Source Data file.

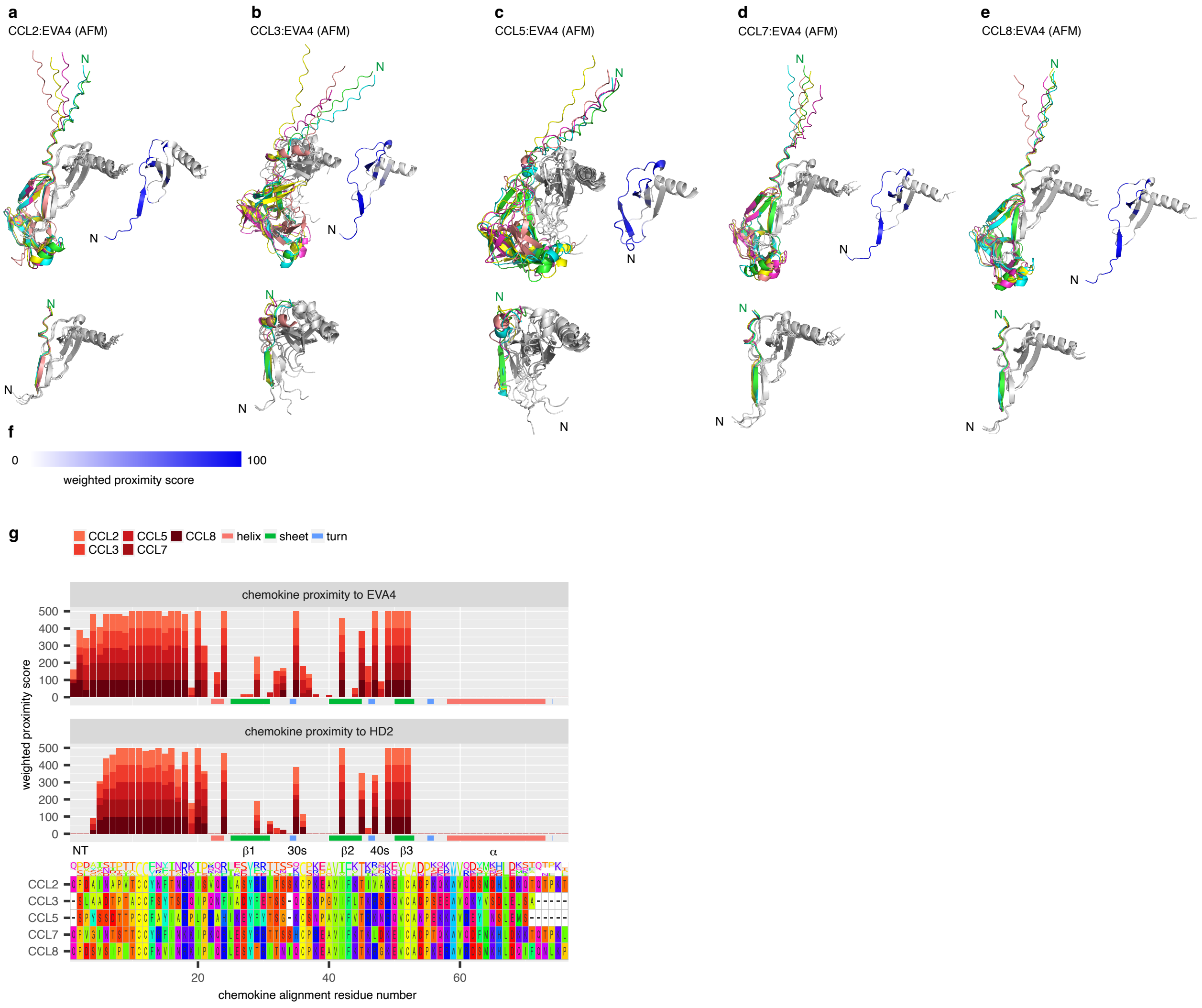

Supplementary Figure 9. Predicted binding modes of EVA4 to chemokines using AlphaFold2-Multimer. a-e, *Left panels:* Ribbon diagrams showing predicted poses for chemokine:EVA4 (*top*) and showing only the HD2 segment within the chemokine:EVA4 model (*bottom*). Chemokines are in gray, and evasin in colour, with the top-ranked pose coloured green. *Right panels:* Corresponding heatmaps of weighted proximity scores mapped onto ribbon diagrams of the chemokine. **f,** Scale bar for weighted proximity score. **g,** Stacked bar charts of weighted proximity scores (y-axis) for EVA4 (*top panel*) and for HD2 (*middle panel*), plotted for each chemokine residue (x-axis) following multiple sequence alignment (*bottom panel*). Models of chemokine:HD2 and chemokine:EVA4 were generated using AlphaFold2-Multimer. Secondary structural elements for CCL8 are indicated at the bottom of the top and middle panels. Chemokines and secondary structural elements are coloured as indicated in the legend. Amino acid residues are coloured using the Taylor scale. *Abbreviation:* N: N-terminal residue of the top-ranked pose. Source data are provided as a Source Data file.

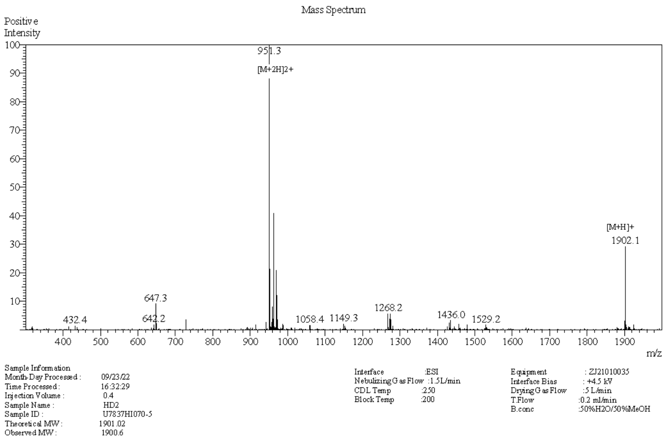

Supplementary Figure 10. Peptide molecular weight. Peptide mass spectrometry data (X-axis m/z ratio, Y-axis positive intensity) provided by Genscript showing theoretical and observed molecular weights of peptide HD2.

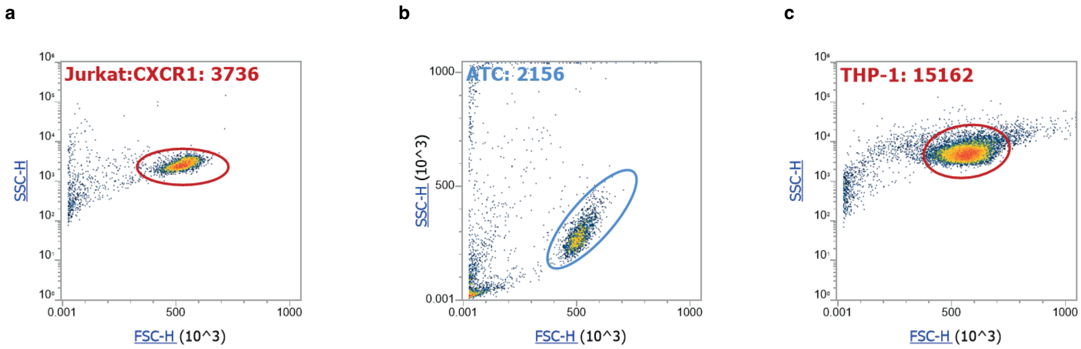

Supplementary Figure 11. Gating strategies for cell counts using flow cytometry. a, Jurkat CXCR1 cells (manuscript figures 5j, 6e, 8a) **b,** Activated T-cells (manuscript figures 5g, h, 6d, 8a), **c,** THP1 cells (manuscript figures 5a-e, 6a-c,g,h, 8a). Pseudo-color dot-plots of SSC-H (side-scatter height y-axis) versus FSC-H (forward-scatter height, x-axis) are shown in each case. The gates are drawn based on previously determined live cell populations and are identical between control and test experiments.

Supplementary Table 1. Human Biotinylated Bait Sources

Bait	Supplier	Supplier Code
C5A	Almac	CB-90
CCL1	Almac	CB-07
CCL11	Almac	CB-03
CCL15	Almac	CB-19
CCL17	Almac	CB-16
CCL18	Almac	CB-21
CCL19	Almac	CB-06
CCL2	Almac	CB-02
CCL20	Almac	CB-05
CCL22	Almac	CB-04
CCL25	Almac	CB-15
CCL28	Almac	CB-20
CCL3	Almac	CB-01
CCL4	Almac	CB-23
CCL5	Almac	CB-08
CCL8	Almac	CB-18
CX3CL1	Almac	CB-14
CXCL1	Protein Foundry	PFP029TBL
CXCL10	Almac	CB-10
CXCL11	Almac	CB-11
CXCL11	Almac	CB-13
CXCL12	Almac	CB-11
CXCL12B	Almac	CB-22
CXCL13	Almac	CB-12
CXCL14	Almac	CB-17
CXCL5	Protein Foundry	PFP004TBL
CXCL8	Almac	CB-09

Supplementary Table 2. Peptide Sequences and Originating Proteins.

Peptide ID	Peptide Sequence	UniProtID:GenBank Accession
HD990	ELNCWHDMPPPVSTDT	AMA01:AEO36603.1
HD2045	KVANRMQGNVTYTCPV	ATR02:A0A023G9N5
HD4578	YDYTEGCPFVVLGNGT	E1180_AMBTT:A0A023G6B6
HD4357	VLKLLGCHYFCNGTLC	E1243_AMBAM:A0A0C9S461
HD540	DEDYEDFFKPVTCYFA	EV672_RHIPC:L7MC74
HD744	DYEDFFKPVTCYFANS	EV672_RHIPC:L7MC74
HD845	EDEDYEDFFKPVTCYF	EV672_RHIPC:L7MC74
HD883	EDYEDFFKPVTCYFAN	EV672_RHIPC:L7MC74
HD1054	ERKVWDRMTPMLWYES	EV974_AMBCJ:A0A023FDY8
HD2312	LTDGTAAYVVERKVWD	EV974_AMBCJ:A0A023FDY8, EV546_AMBCJ:A0A023FBW7
HD2313	LTDGTACYVVERKVWD	EV974_AMBCJ:A0A023FDY8, EV546_AMBCJ:A0A023FBW7
HD2314	LTDGTASYVVERKVWD	EV974_AMBCJ:A0A023FDY8, EV546_AMBCJ:A0A023FBW7
HD3838	TDGTACYVVERKVWDR	EV974_AMBCJ:A0A023FDY8, EV546_AMBCJ:A0A023FBW7
HD3839	TDGTASYVVERKVWDR	EV974_AMBCJ:A0A023FDY8, EV546_AMBCJ:A0A023FBW7
HD4670	YPCYNLTEHQAKNLTT	EV991_AMBCJ:A0A023FFD0
HD2	EEDDYTAYAPLTCYFT	EVA4_RHISA:P0C8E9
HD2133	LEEEDDYTAYAPLTAY	EVA4_RHISA:P0C8E9
HD2134	LEEEDDYTAYAPLTCY	EVA4_RHISA:P0C8E9
HD2135	LEEEDDYTAYAPLTSY	EVA4_RHISA:P0C8E9
HD534	DDYTAYAPLTCYFTNS	EVA4_RHISA:P0C8E9
HD612	DLEEEDDYTAYAPLTS	EVA4_RHISA:P0C8E9
HD7	EEEDDYTAYAPLTCYF	EVA4_RHISA:P0C8E9
HD842	EDDYTAYAPLTCYFTN	EVA4_RHISA:P0C8E9
HD900	EEDDYTAYAPLTAYFT	EVA4_RHISA:P0C8E9
HD901	EEDDYTAYAPLTSYFT	EVA4_RHISA:P0C8E9
HD908	EEEDDYTAYAPLTAYF	EVA4_RHISA:P0C8E9
HD909	EEEDDYTAYAPLTSYF	EVA4_RHISA:P0C8E9
HD1267	GAPVVGIGGLDNKTWH	IHO01:A0A223FZ07

Peptide ID	Peptide Sequence	UniProtID:GenBank Accession
HD3398	SCVAKVDSLSKEEATV	IRI01:JAA70749.1
HD4650	YNIVITKNKTLVVNCT	RPU02:JAA60771.1

Supplementary Table 3. Human Chemokine Panel Sources. BLI = Biolayer Interferometry, C = Chemotaxis

Chemokine	Supplier	Supplier Code	Assay
CCL1	Peprotech	300-37	BLI
CCL11	Peprotech	300-21	BLI
CCL13	Peprotech	300-24	BLI
CCL14	Peprotech	300-38	BLI
CCL14	Peprotech	300-38B	C
CCL15	Novus	NBP2-35043	C, BLI
CCL16	Peprotech	300-44	BLI
CCL17	Peprotech	300-30	BLI
CCL18	Peprotech	300-34	BLI
CCL19	Peprotech	300-29B	BLI
CCL2	Peprotech	300-04	C, BLI
CCL20	Peprotech	300-29A	BLI
CCL21	Peprotech	300-35A	BLI
CCL22	Peprotech	300-36A	BLI
CCL23	Biolegend	587002	C, BLI
CCL24	Peprotech	300-33	BLI
CCL25	Peprotech	300-45	BLI
CCL26	Peprotech	300-48	BLI
CCL27	Peprotech	300-54	BLI
CCL28	Peprotech	300-57	BLI
CCL3	Peprotech	300-08	C, BLI
CCL3L1	Peprotech	300-56	BLI
CCL4	Peprotech	300-09	BLI
CCL4L1	Peprotech	300-58	BLI
CCL5	Peprotech	300-06	C, BLI
CCL7	Peprotech	300-17	C, BLI
CCL8	Peprotech	300-15	C, BLI
CX3CL1	Peprotech	300-31	BLI
CXCL1	Peprotech	300-11	BLI
CXCL10	Peprotech	300-12	C, BLI

Chemokine	Supplier	Supplier Code	Assay
CXCL11	Peprotech	300-46	C, BLI
CXCL12	Peprotech	300-28A	BLI
CXCL12B	Peprotech	300-28B	BLI
CXCL13	Peprotech	300-47	BLI
CXCL14	Peprotech	300-50	BLI
CXCL16	Peprotech	300-55	BLI
CXCL2	Peprotech	300-39	BLI
CXCL3	Peprotech	300-40	BLI
CXCL4	Peprotech	300-16	BLI
CXCL5	Peprotech	300-22	BLI
CXCL6	Peprotech	300-41	C, BLI
CXCL7	Peprotech	300-14	BLI
CXCL8	Peprotech	200-08	BLI
CXCL9	Peprotech	300-26	BLI

Supplementary Table 4. Biolayer Interferometry Analysis. Expt ID indicates independent experiments. K_D[M]: Mean K_D (molar). SE: standard error. N: number of dose responses with good fits used to calculate summary statistics. sHD2 is SUMO:HD2.

Expt ID	Sample	Analyte	K_D [M]	K_D:SE [M]	N
E12	sHD2	CCL8	1.425e-07	4.94e-08	5
E32	sHD2	CCL8	8.49e-08	1.90e-08	4

Supplementary Table 5. Exact P values for phage-display mutagenesis analyses of scanning mutants, comparisons to HD2 as negative control

Residue	Substitution type	Number of samples	P value
E2	alanine	24	8.46e-03
D3	alanine	24	3.42e-05
D4	alanine	24	1.01e-10
Y5	alanine	24	2.93e-11
Y8	alanine	24	2.34e-11
P10	alanine	24	3.49e-13
L11	alanine	24	1.71e-02
T12	alanine	24	1.97e-03
C13	alanine	24	4.44e-16
Y14	alanine	24	3.94e-11
Y5	anionic	48	4.64e-04
T6	anionic	48	9.19e-05
A7	anionic	48	3.82e-02
Y8	anionic	48	1.22e-11
A9	anionic	48	2.79e-06
P10	anionic	48	3.12e-09
L11	anionic	48	4.37e-13
T12	anionic	48	1.29e-07
C13	anionic	48	0.00e+00
Y14	anionic	48	1.39e-11
F15	anionic	48	1.69e-05
E1	arginine	24	1.31e-10
E2	arginine	24	3.83e-09
D3	arginine	24	6.86e-14
D4	arginine	24	3.95e-13
Y5	arginine	24	1.89e-15
Y8	arginine	24	0.00e+00
A9	arginine	24	6.47e-06
P10	arginine	24	0.00e+00
T12	arginine	24	1.84e-04

Residue	Substitution type	Number of samples	P value
C13	arginine	24	0.00e+00
Y14	arginine	24	0.00e+00
F15	arginine	24	7.50e-03
T16	arginine	24	1.84e-05
Y5	aspartate	24	4.12e-08
T6	aspartate	24	3.57e-05
A7	aspartate	24	3.75e-02
Y8	aspartate	24	4.76e-11
A9	aspartate	24	0.00e+00
P10	aspartate	24	9.42e-07
L11	aspartate	24	3.66e-15
T12	aspartate	24	5.40e-08
C13	aspartate	24	1.11e-16
Y14	aspartate	24	2.87e-09
F15	aspartate	24	7.02e-06
E1	cationic	48	4.50e-12
E2	cationic	48	2.38e-08
D3	cationic	48	2.35e-14
D4	cationic	48	0.00e+00
Y5	cationic	48	0.00e+00
T6	cationic	48	3.34e-02
Y8	cationic	48	0.00e+00
A9	cationic	48	4.93e-11
P10	cationic	48	0.00e+00
L11	cationic	48	2.60e-02
T12	cationic	48	6.56e-08
C13	cationic	48	0.00e+00
Y14	cationic	48	0.00e+00
F15	cationic	48	1.96e-04
T16	cationic	48	1.22e-04
E1D	conservative	24	4.79e-02

Residue	Substitution type	Number of samples	P value
A9G	conservative	24	3.50e-12
P10A	conservative	24	1.11e-16
T12A	conservative	24	3.21e-04
C13S	conservative	24	6.10e-10
T6	glutamate	24	2.30e-03
Y8	glutamate	24	6.47e-09
P10	glutamate	24	6.49e-09
L11	glutamate	24	4.62e-07
T12	glutamate	24	5.56e-05
C13	glutamate	24	3.57e-13
Y14	glutamate	24	1.15e-09
F15	glutamate	24	1.66e-03
E1	hydrophobic	96	1.27e-02
E2	hydrophobic	96	3.24e-03
D3	hydrophobic	96	8.93e-14
D4	hydrophobic	96	0.00e+00
Y5	hydrophobic	96	2.73e-10
T6	hydrophobic	96	8.30e-04
Y8	hydrophobic	96	0.00e+00
A9	hydrophobic	96	0.00e+00
P10	hydrophobic	96	0.00e+00
L11	hydrophobic	72	7.66e-03
C13	hydrophobic	96	2.22e-16
Y14	hydrophobic	96	1.32e-09
E2	isoleucine	24	4.22e-02
D3	isoleucine	24	2.52e-09
D4	isoleucine	24	2.55e-11
Y5	isoleucine	24	1.11e-09
T6	isoleucine	24	5.00e-09
Y8	isoleucine	24	0.00e+00
A9	isoleucine	24	0.00e+00

Residue	Substitution type	Number of samples	P value
P10	isoleucine	24	0.00e+00
L11	isoleucine	24	3.20e-03
C13	isoleucine	24	1.85e-10
Y14	isoleucine	24	9.15e-05
E2	leucine	24	3.37e-02
D3	leucine	24	1.14e-07
D4	leucine	24	2.66e-09
Y5	leucine	24	1.08e-02
Y8	leucine	24	6.68e-13
A9	leucine	24	9.59e-09
P10	leucine	24	0.00e+00
C13	leucine	24	5.55e-16
Y14	leucine	24	3.43e-09
E1	lysine	24	7.73e-08
E2	lysine	24	3.38e-06
D3	lysine	24	3.74e-08
D4	lysine	24	2.66e-15
Y5	lysine	24	4.61e-11
T6	lysine	24	2.04e-04
Y8	lysine	24	9.76e-14
A9	lysine	24	4.71e-11
P10	lysine	24	0.00e+00
L11	lysine	24	3.04e-03
T12	lysine	24	1.22e-08
C13	lysine	24	0.00e+00
Y14	lysine	24	0.00e+00
F15	lysine	24	2.93e-04
T16	lysine	24	3.09e-02
E2	methionine	24	6.31e-03
D3	methionine	24	4.57e-07
D4	methionine	24	8.36e-10

Residue	Substitution type	Number of samples	P value
Y5	methionine	24	5.95e-04
Y8	methionine	24	8.44e-14
A9	methionine	24	2.73e-05
P10	methionine	24	1.22e-15
C13	methionine	24	8.84e-11
Y14	methionine	24	1.21e-06
E1	valine	24	1.37e-03
D3	valine	24	8.51e-12
D4	valine	24	5.66e-14
Y5	valine	24	1.49e-13
T6	valine	24	1.24e-05
Y8	valine	24	1.01e-08
A9	valine	24	9.38e-14
P10	valine	24	0.00e+00
C13	valine	24	4.44e-07
Y14	valine	24	9.37e-06

Supplementary Table 6. Exact P values for cell migration experiments with alanine-scanning mutants, comparisons to HD2 as negative control

Experiment components	Number of samples	P value
THP1, CCL5, HD2_D4A	3	1.65e-02
THP1, CCL5, HD2_Y5A	3	7.00e-06
THP1, CCL5, HD2_Y8A	3	2.24e-04
THP1, CCL5, HD2_P10A	3	0.00e+00
THP1, CCL5, HD2_C13A	3	0.00e+00
THP1, CCL5, HD2_Y14A	3	3.29e-02
THP1, CCL5, HD2SCR	3	0.00e+00
THP1, CCL7, HD2_E1A	3	5.61e-03
THP1, CCL7, HD2_E2A	3	3.63e-02
THP1, CCL7, HD2_D4A	3	2.94e-03
THP1, CCL7, HD2_Y5A	3	3.70e-05
THP1, CCL7, HD2_Y8A	3	1.30e-05
THP1, CCL7, HD2_P10A	3	3.19e-03
THP1, CCL7, HD2_C13A	3	2.12e-03
THP1, CCL7, HD2_Y14A	3	1.37e-04
THP1, CCL7, HD2SCR	3	2.50e-05
THP1, CCL8, HD2_P10A	3	1.30e-05
THP1, CCL8, HD2SCR	3	4.56e-04
ATC, CXCL10, HD2_L11A	3	8.00e-06
ATC, CXCL10, HD2_C13A	3	0.00e+00
ATC, CXCL10, HD2_Y14A	3	2.35e-04
ATC, CXCL10, HD2_F15A	3	1.43e-03
ATC, CXCL10, HD2SCR	3	0.00e+00
J:CXCR1, CXCL6, HD2_P10A	3	1.19e-02
J:CXCR1, CXCL6, HD2_L11A	3	1.00e-06
J:CXCR1, CXCL6, HD2_C13A	3	5.80e-05
J:CXCR1, CXCL6, HD2SCR	3	2.00e-06

Supplementary Table 7. Rosetta cross-interface binding energy for chemokine:HD2 peptide complexes. dG_cross = cross-interface binding energy, kcal/mol; ADCP = AutoDock Crankpep; AFM = AlphaFold2-Multimer.

Method	Chemokine	Rank	dG_cross
ADCP	CCL2	1	-66.579
ADCP	CCL2	2	-76.181
ADCP	CCL2	3	-76.421
ADCP	CCL2	4	-70.362
ADCP	CCL2	5	-64.921
ADCP	CCL2	6	-64.931
ADCP	CCL2	7	-72.182
ADCP	CCL2	8	-52.433
ADCP	CCL2	9	-67.718
ADCP	CCL2	10	-59.412
ADCP	CCL3	1	-63.724
ADCP	CCL3	2	-80.354
ADCP	CCL3	3	-63.587
ADCP	CCL3	4	-73.507
ADCP	CCL3	5	-66.313
ADCP	CCL3	6	-50.390
ADCP	CCL3	7	-56.006
ADCP	CCL3	8	-66.245
ADCP	CCL3	9	-61.101
ADCP	CCL3	10	-45.779
ADCP	CCL5	1	-61.450
ADCP	CCL5	2	-52.291
ADCP	CCL5	3	-74.971
ADCP	CCL5	4	-66.801
ADCP	CCL5	5	-61.093
ADCP	CCL5	6	-59.012
ADCP	CCL5	7	-67.321
ADCP	CCL5	8	-54.052
ADCP	CCL5	9	-64.403
ADCP	CCL5	10	-73.192

Method	Chemokine	Rank	dG_cross
ADCP	CCL7	1	-78.344
ADCP	CCL7	2	-44.944
ADCP	CCL7	3	-54.433
ADCP	CCL7	4	-62.307
ADCP	CCL7	5	-62.046
ADCP	CCL7	6	-59.925
ADCP	CCL7	7	-60.735
ADCP	CCL7	8	-57.171
ADCP	CCL7	9	-53.523
ADCP	CCL7	10	-57.840
ADCP	CCL8	1	-78.777
ADCP	CCL8	2	-57.479
ADCP	CCL8	3	-59.036
ADCP	CCL8	4	-43.707
ADCP	CCL8	5	-46.076
ADCP	CCL8	6	-59.917
ADCP	CCL8	7	-63.846
ADCP	CCL8	8	-59.833
ADCP	CCL8	9	-65.773
ADCP	CCL8	10	-68.998
ADCP	CXCL10	1	-59.657
ADCP	CXCL10	2	-49.184
ADCP	CXCL10	3	-60.169
ADCP	CXCL10	4	-55.894
ADCP	CXCL10	5	-39.994
ADCP	CXCL10	6	-52.237
ADCP	CXCL10	7	-42.611
ADCP	CXCL10	8	-54.275
ADCP	CXCL10	9	-49.530
ADCP	CXCL10	10	-65.629
ADCP	CXCL11	1	-54.691
ADCP	CXCL11	2	-47.716

Method	Chemokine	Rank	dG_cross
ADCP	CXCL11	3	-58.574
ADCP	CXCL11	4	-60.050
ADCP	CXCL11	5	-56.787
ADCP	CXCL11	6	-47.383
ADCP	CXCL11	7	-51.849
ADCP	CXCL11	8	-48.870
ADCP	CXCL11	9	-54.059
ADCP	CXCL11	10	-51.668
ADCP	CXCL6	1	-62.304
ADCP	CXCL6	2	-68.005
ADCP	CXCL6	3	-57.081
ADCP	CXCL6	4	-56.619
ADCP	CXCL6	5	-64.228
ADCP	CXCL6	6	-58.329
ADCP	CXCL6	7	-50.751
ADCP	CXCL6	8	-50.345
ADCP	CXCL6	9	-62.787
ADCP	CXCL6	10	-59.142
AFM	CCL2	0	-61.016
AFM	CCL2	1	-66.362
AFM	CCL2	2	-62.620
AFM	CCL2	3	-56.150
AFM	CCL2	4	-57.081
AFM	CCL3	0	-66.913
AFM	CCL3	1	-55.862
AFM	CCL3	2	-52.177
AFM	CCL3	3	-49.282
AFM	CCL3	4	-44.948
AFM	CCL5	0	-56.264
AFM	CCL5	1	-51.722
AFM	CCL5	2	-48.543
AFM	CCL5	3	-43.941

Method	Chemokine	Rank	dG_cross
AFM	CCL5	4	-61.443
AFM	CCL7	0	-64.503
AFM	CCL7	1	-63.382
AFM	CCL7	2	-65.303
AFM	CCL7	3	-69.799
AFM	CCL7	4	-63.075
AFM	CCL8	0	-70.178
AFM	CCL8	1	-72.505
AFM	CCL8	2	-76.024
AFM	CCL8	3	-77.569
AFM	CCL8	4	-74.654
AFM	CXCL10	0	-57.988
AFM	CXCL10	1	-60.566
AFM	CXCL10	2	-56.348
AFM	CXCL10	3	-51.332
AFM	CXCL10	4	-57.651
AFM	CXCL11	0	-53.576
AFM	CXCL11	1	-66.151
AFM	CXCL11	2	-20.341
AFM	CXCL11	3	-3.475
AFM	CXCL11	4	-19.678
AFM	CXCL6	0	-60.062
AFM	CXCL6	1	-62.350
AFM	CXCL6	2	-53.337
AFM	CXCL6	3	-68.376
AFM	CXCL6	4	-58.957
