# Supplementary material for: Discovery and pharmacophoric characterization of chemokine network inhibitors using phage-display, saturation mutagenesis and computational modelling
Source: Nat Commun. 2023 Sep 16;14:5763. doi: 10.1038/s41467-023-41488-z (PMC10505172; doi:10.1038/s41467-023-41488-z)
Supplement: Supplementary file 3 — Reporting Summary [file 41467_2023_41488_MOESM3_ESM.pdf]

nature portfolio

Corresponding author(s): Shoumo Bhattacharya
Last updated by author(s): Aug 9, 2023

Reporting Summary

Nature Portfolio wishes to improve the reproducibility of the work that we publish. This form provides structure for consistency and transparency in reporting. For further information on Nature Portfolio policies, see our Editorial Policies and the Editorial Policy Checklist.

Statistics

For all statistical analyses, confirm that the following items are present in the figure legend, table legend, main text, or Methods section.

n/a	Confirmed	
☐	☒	The exact sample size (*n*) for each experimental group/condition, given as a discrete number and unit of measurement
☐	☒	A statement on whether measurements were taken from distinct samples or whether the same sample was measured repeatedly
☐	☒	The statistical test(s) used AND whether they are one- or two-sided<br>*Only common tests should be described solely by name; describe more complex techniques in the Methods section.*
☒	☐	A description of all covariates tested
☐	☒	A description of any assumptions or corrections, such as tests of normality and adjustment for multiple comparisons
☐	☒	A full description of the statistical parameters including central tendency (e.g. means) or other basic estimates (e.g. regression coefficient) AND variation (e.g. standard deviation) or associated estimates of uncertainty (e.g. confidence intervals)
☐	☒	For null hypothesis testing, the test statistic (e.g. *F*, *t*, *r*) with confidence intervals, effect sizes, degrees of freedom and *P* value noted<br>*Give P values as exact values whenever suitable.*
☒	☐	For Bayesian analysis, information on the choice of priors and Markov chain Monte Carlo settings
☒	☐	For hierarchical and complex designs, identification of the appropriate level for tests and full reporting of outcomes
☒	☐	Estimates of effect sizes (e.g. Cohen's *d*, Pearson's *r*), indicating how they were calculated

Our web collection on statistics for biologists contains articles on many of the points above.

Software and code

Policy information about availability of computer code

Data collection	Biolayer interferometry data was collected and analyzed using ForteBio Data Analysis HT 11.1. Flow sorting data was collected and analyzed using Attune Cytometric Software v5.1.1. Structural models were generated using AlphaFold2-Multimer at the COSMIC2 Science Gateway49, 73, and ADFRSuite 1.050 (https://ccsb.scripps.edu/adcp/downloads/) and reduce ((https://github.com/rlabduke/reduce) on Oxford University BioMedical Research Computing Cluster (BMRCC).
Data analysis	Data analysis was performed using R version 4.2.3, RStudio version 2023.03.2+454, running on arch64-apple-darwin20 (64-bit) with macOS Ventura 13.4.1. R packages used were: ape_5.7-1, asbio_1.9-2, bio3d_2.4-4, Biobase_2.58.0, BiocGenerics_0.44.0, BiocParallel_1.32.6, Biostrings_2.66.0, dendsort_0.3.4, DescTools_0.99.48, dplyr_1.1.2, drc_3.0-1, flextable_0.9.1, forcats_1.0.0, GenomeInfoDb_1.34.9, GenomicAlignments_1.34.1, GenomicRanges_1.50.2, ggmsa_1.4.0, ggnewscale_0.4.8, ggplot2_3.4.2, ggpubr_0.6.0, ggseqlogo_0.1, ggtree_3.6.2, ggupset_0.3.0, gridExtra_2.3, gtable_0.3.3, IRanges_2.32.0, janitor_2.2.0, jsonlite_1.8.4, lubridate_1.9.2, maps_3.4.1, MASS_7.3-58.2, MatrixGenerics_1.10.0, matrixStats_0.63.0, msa_1.30.1, officer_0.6.2, openxlsx_4.2.5.2, pals_1.7, pheatmap_1.0.12, phytools_1.5-1, purrr_1.0.1, R.methodsS3_1.8.2, R.oo_1.25.0, R.utils_2.12.2, RColorBrewer_1.1-3, readr_2.1.4, Rsamtools_2.14.0, S4Vectors_0.36.2, scales_1.2.1, seqinr_4.2-30, ShortRead_1.56.1, strex_1.6.0, stringr_1.5.0, SummarizedExperiment_1.28.0, tibble_3.2.1, tidyr_1.3.0, tidyverse_2.0.0, viridis_0.6.3, viridisLite_0.4.1, XVector_0.38.0, zoo_1.8-12. Arpeggio analysis used Python 2.7, biopython 1.79, and Arpeggio62 (https://bitbucket.org/harryjubb/arpeggio/src/master/). Open-Source PyMOL (https://pymolwiki.org/index.php/MAC_Install) was used for scripting and PyMOL 2.5.2 (https://pymol.org/2/) was used for visualization of structural models. Custom scripts are provided as source data.

For manuscripts utilizing custom algorithms or software that are central to the research but not yet described in published literature, software must be made available to editors and reviewers. We strongly encourage code deposition in a community repository (e.g. GitHub). See the Nature Portfolio guidelines for submitting code & software for further information.

Data

Policy information about availability of data

All manuscripts must include a data availability statement. This statement should provide the following information, where applicable:

- Accession codes, unique identifiers, or web links for publicly available datasets
- A description of any restrictions on data availability
- For clinical datasets or third party data, please ensure that the statement adheres to our policy

The authors declare that the data supporting the findings of this study are available within the paper and its supplementary information files. Source data are provided with this paper. Plasmids described and sequences are available on request from the corresponding author.

Research involving human participants, their data, or biological material

Policy information about studies with human participants or human data. See also policy information about sex, gender (identity/presentation), and sexual orientation and race, ethnicity and racism.

Reporting on sex and gender	This is not applicable as anonymised samples were obtained from NHSBT
Reporting on race, ethnicity, or other socially relevant groupings	This is not applicable as anonymised samples were obtained from NHSBT
Population characteristics	This is not applicable as anonymised samples were obtained from NHSBT
Recruitment	This is not applicable as anonymised samples were obtained from NHSBT
Ethics oversight	MEDICAL SCIENCES INTERDIVISIONAL RESEARCH ETHICS COMMITTEE, UNIVERSITY OF OXFORD

Note that full information on the approval of the study protocol must also be provided in the manuscript.

Field-specific reporting

Please select the one below that is the best fit for your research. If you are not sure, read the appropriate sections before making your selection.

☒ Life sciences ☐ Behavioural & social sciences ☐ Ecological, evolutionary & environmental sciences

For a reference copy of the document with all sections, see nature.com/documents/nr-reporting-summary-flat.pdf

Life sciences study design

All studies must disclose on these points even when the disclosure is negative.

Sample size	For cell-based experiments sample size was not formally calculated. Cell numbers, numbers of technical and biological replicates are based on optimization of such experiments 20-23, 33.
Data exclusions	None
Replication	Typically n=3 technical replicates for each of n=3 biological replicates. Replicates were deemed unsuccessful if the positive control within the experiment did not show the expected response.
Randomization	None. This is not relevant to cell - based studies.
Blinding	Investigators were blinded at analysis to group allocation, but not at data collection. Blinding is not relevant in high-throughput data collection experiments where an automated instrument (E.g. Octet for BLI, or Attune for cell-migration) collects data.

Reporting for specific materials, systems and methods

We require information from authors about some types of materials, experimental systems and methods used in many studies. Here, indicate whether each material, system or method listed is relevant to your study. If you are not sure if a list item applies to your research, read the appropriate section before selecting a response.

Materials & experimental systems

n/a	Involved in the study
☒	☐ Antibodies
☐	☒ Eukaryotic cell lines
☒	☐ Palaeontology and archaeology
☒	☐ Animals and other organisms
☒	☐ Clinical data
☒	☐ Dual use research of concern
☒	☐ Plants

Methods

n/a	Involved in the study
☒	☐ ChIP-seq
☐	☒ Flow cytometry
☒	☐ MRI-based neuroimaging

Eukaryotic cell lines

Policy information about cell lines and Sex and Gender in Research

Cell line source(s)	THP1 cells were obtained from ECACC and Jurkat cells were obtained from ATCC
Authentication	None
Mycoplasma contamination	All cell lines are tested monthly for mycoplama and tested negative.
Commonly misidentified lines (See ICLAC register)	None.

Flow Cytometry

Plots

Confirm that:

☒ The axis labels state the marker and fluorochrome used (e.g. CD4-FITC).

☒ The axis scales are clearly visible. Include numbers along axes only for bottom left plot of group (a 'group' is an analysis of identical markers).

☒ All plots are contour plots with outliers or pseudocolor plots.

☒ A numerical value for number of cells or percentage (with statistics) is provided.

Methodology

Sample preparation	Not applicable as used for counting cells
Instrument	ATTUNE NxT
Software	Attune Cytometric Software v5.1.1
Cell population abundance	Forward and side scatter
Gating strategy	Forward and side scatter

☒ Tick this box to confirm that a figure exemplifying the gating strategy is provided in the Supplementary Information.
